# The impact of body temperature abnormalities on the disease severity and outcome in patients with severe sepsis: an analysis from a multicenter, prospective survey of severe sepsis

**DOI:** 10.1186/cc13106

**Published:** 2013-11-13

**Authors:** Shigeki Kushimoto, Satoshi Gando, Daizoh Saitoh, Toshihiko Mayumi, Hiroshi Ogura, Seitaro Fujishima, Tsunetoshi Araki, Hiroto Ikeda, Joji Kotani, Yasuo Miki, Shin-ichiro Shiraishi, Koichiro Suzuki, Yasushi Suzuki, Naoshi Takeyama, Kiyotsugu Takuma, Ryosuke Tsuruta, Yoshihiro Yamaguchi, Norio Yamashita, Naoki Aikawa

**Affiliations:** 1 Division of Emergency Medicine, Tohoku University Graduate School of Medicine, Seiryo-machi 1-1, Aoba-ku, Sendai, Miyagi 980-8574, Japan; 2 Division of Acute and Critical Care Medicine, Department of Anesthesiology and Critical Care Medicine, Hokkaido University Graduate School of Medicine, Kita 2-jou Nishi 5, Kitaku, Sapporo-shi, Hokkaido 060-8638, Japan; 3 Division of Traumatology, Research Institute, National Defense Medical College, 3-2 Namiki, Tokorozawa-shi, Saitama 359-8513, Japan; 4 Emergency Center, Department of Emergency and Critical Care Medicine, Ichinomiya Municipal Hospital, Bunkyo 2-2-22, Ichinomiya-shi, Aichi 491-8558, Japan; 5 Department of Traumatology and Acute Critical Medicine, Osaka University Graduate School of Medicine, 2-15 Yamadaoka, Suita-shi, Osaka 565-0871, Japan; 6 Department of Emergency & Critical Care Medicine, School of Medicine, Keio University, Shinanomachi 35, Shinjukuku, Tokyo 160-8582, Japan; 7 Department of Emergency & Critical Care Medicine, Trauma Center St. Mary’s Hospital, Tsubukuhonmachi 422, Kurume-shi, Fukuoka 830-8543, Japan; 8 Department of Emergency Medicine, Trauma and Resuscitation Center, Teikyo University School of Medicine, Kaga 2-11-1, Itabashiku, Tokyo 173-8606, Japan; 9 Department of Emergency, Critical Care and Disaster Medicine, Hyogo College of Medicine, Mukogawa-cho 1-1, Nishinomiya-shi, Hyogo 663-8501, Japan; 10 Advanced Critical Care Center Aichi Medical University Hospital, Yazakokarimata 1-1, Nagakute-shi, Aichi 480-1195, Japan; 11 Department of Emergency and Critical Care Medicine, Nippon Medical School, Sendagi 1-1-5, Bunkyouku, Tokyo 113-8603, Japan; 12 Department of Acute Medicine, Kawasaki Medical School, Matsushima 577, Kurashiki-shi, Okayama 701-0114, Japan; 13 Department of Critical Care Medicine, Iwate Medical University, Uchimaru 19-1, Morioka-shi, Iwate 020-8505, Japan; 14 Department of Emergency and Acute Intensive Care Medicine, Fujita Health University, Dengakugakubo 1-98, Kutsukake-cho, Toyoake-shi, Aichi 470-1192, Japan; 15 Emergency & Critical Care Center, Kawasaki Municipal Hospital, Shinkawadori 12-1, Kawasakiku, Kawasaki-shi, Kanagawa 210-0013, Japan; 16 Advanced Medical Emergency & Critical Care Center, Yamaguchi University Hospital, Minamikogushi 1-1-1, Ube-shi, Yamaguchi 755-8505, Japan; 17 Department of Trauma & Critical Care Medicine, Kyorin University, School of Medicine, Shinkawa 6-20-2, Mitaka-shi, Tokyo 181-8611, Japan; 18 Department of Emergency & Critical Care Medicine, School of Medicine, Kurume University, Asahimachi 67, Kurume-shi, Fukuoka 830-0011, Japan

## Abstract

**Introduction:**

Abnormal body temperatures (T_b_) are frequently seen in patients with severe sepsis. However, the relationship between T_b_ abnormalities and the severity of disease is not clear. This study investigated the impact of T_b_ on disease severity and outcomes in patients with severe sepsis.

**Methods:**

We enrolled 624 patients with severe sepsis and grouped them into 6 categories according to their T_b_ at the time of enrollment. The temperature categories (≤35.5°C, 35.6–36.5°C, 36.6–37.5°C, 37.6–38.5°C, 38.6–39.5°C, ≥39.6°C) were based on the temperature data of the Acute Physiology and Chronic Health Evaluation II (APACHE II) scoring. We compared patient characteristics, physiological data, and mortality between groups.

**Results:**

Patients with T_b_ of ≤36.5°C had significantly worse sequential organ failure assessment (SOFA) scores when compared with patients with T_b_ >37.5°C on the day of enrollment. Scores for APACHE II were also higher in patients with T_b_ ≤35.5°C when compared with patients with T_b_ >36.5°C. The 28-day and hospital mortality was significantly higher in patients with T_b_ ≤36.5°C. The difference in mortality rate was especially noticeable when patients with T_b_ ≤35.5°C were compared with patients who had T_b_ of >36.5°C. Although mortality did not relate to T_b_ ranges of ≥37.6°C as compared to reference range of 36.6–37.5°C, relative risk for 28-day mortality was significantly greater in patients with 35.6–36.5°C and ≤35.5°C (odds ratio; 2.032, 3.096, respectively). When patients were divided into groups based on the presence (≤36.5°C, n = 160) or absence (>36.5°C, n = 464) of hypothermia, disseminated intravascular coagulation (DIC) as well as SOFA and APACHE II scores were significantly higher in patients with hypothermia. Patients with hypothermia had significantly higher 28-day and hospital mortality rates than those without hypothermia (38.1% vs. 17.9% and 49.4% vs. 22.6%, respectively). The presence of hypothermia was an independent predictor of 28-day mortality, and the differences between patients with and without hypothermia were observed irrespective of the presence of septic shock.

**Conclusions:**

In patients with severe sepsis, hypothermia (T_b_ ≤36.5°C) was associated with increased mortality and organ failure, irrespective of the presence of septic shock.

**Trial registration:**

UMIN-CTR ID UMIN000008195

## Introduction

Body temperature (T_b_) abnormalities are amongst the most commonly noted symptoms of critically ill patients. Fever occurs in approximately half of patients admitted to the ICU and has been associated with adverse outcomes [1]. Fever is one of the most prominent symptoms of infection [2] and it is part of the host acute-phase response to infectious as well as non-infectious inflammatory stimuli [3]. Fever is also believed to be harmful, especially in patients with life-threatening illness, because febrile responses are known to increase the metabolic rate and minutes ventilation and oxygen consumption, and it can have adverse effects on neurological outcomes [4-6]. Fever could also be beneficial because it is believed to reduce bacterial growth, and a higher T_b_ is believed to promote the synthesis of antibodies and cytokines, thereby activating immune cells and improving survival [7-9]. Several studies have suggested that suppression of the febrile response with antipyretic drugs could worsen patient outcomes [10,11].

A large epidemiological study that included patients with and without infection reported that the presence of fever per se could not be associated with increased ICU mortality. Nevertheless, fever with T_b_ ≥39.5°C was associated with a significant increase in mortality (20.3% versus 12.0% (*P* <0.001) for patients with T_b_ ≥39.5°C and <39.5°C, respectively). These very high fevers could be complicated with cardiac arrhythmias, tachycardia, increased oxygen demand, convulsions, and brain damage [1]. A recent study that used data from Australia, New Zealand, and the United Kingdom investigated the association between peak T_b_ in the first 24 h after admission to ICU and in-hospital mortality [12]. This study showed that elevated peak T_b_ in the first 24 h in the ICU could be associated with decreased in-hospital mortality in patients with infection. The lowest mortality risk was among patients with T_b_ between 39.0°C and 39.4°C. However, mortality risk was increased among patients who did not have infection. Patients with fever in response to non-infective causes may well experience the harmful effects of fever without any fever-related benefits, such as protection against viruses or bacteria.

Hypothermia can be caused by a variety of factors including cold exposure, severe infection, endocrine abnormalities, and drug overdoses, and hypothermic patients require immediate medical intervention [13-15]. Although hypothermia may be an unintended consequence of critical illness and may be associated with an increased risk of mortality in patients with sepsis and non-infectious conditions, the influence of hypothermia on the physiological severity and outcome of critically ill patients, particularly patients with severe sepsis, is not well understood [12,16-22].

Although there are many reports of T_b_ abnormalities in patients with sepsis, there is a relative paucity of information on the influences of hyper- or hypothermia on disease severity and outcomes in patients with severe sepsis. The aim of present study was to investigate the association between T_b_ and disease severity and patient outcomes in patients with a definitive diagnosis of severe sepsis.

## Materials and methods

This was a prospective study conducted as a part of a multicenter prospective evaluation of severe sepsis in Japan, undertaken by the Japanese Association for Acute Medicine Sepsis Registry (JAAMSR) Study Group [23]. Both the Japanese Association for Acute Medicine and the Ethics Committees of the hospitals that participated in this study approved the study protocol. Data collection was performed as part of the routine clinical examinations without any medical intervention. Data management and statistical analyses were processed anonymously. Based on these reasons, written informed consent was waived by both the Japanese Association for Acute Medicine and the Ethics Committees of the participating hospitals. The study was registered with the University Hospital Medical Information Network Clinical Trials Registry (UMIN-CTR ID: UMIN000008195).

### Patients

Between June 1, 2010, and May 31, 2011, we enrolled 624 patients in this study. All of the patients were diagnosed with severe sepsis and admitted to one of 15 critical care centers in the tertiary care hospitals in Japan. We did not have any exclusion criteria.

### Definitions

Sepsis, severe sepsis, septic shock, and systemic inflammatory response syndrome (SIRS) were defined according to the American College of Chest Physicians/Society of Critical Care Medicine consensus conference and its revised version of 2003 [24,25]. The severity of illness was evaluated according to the acute physiology and chronic health evaluation (APACHE) II score at the time of enrollment [26]. Organ dysfunction was assessed according to the sequential organ failure assessment (SOFA) score [27]. Multiple organ dysfunction syndrome (MODS) was defined as a SOFA score ≥12 [27]. A diagnosis of disseminated intravascular coagulation (DIC) was made on the basis of the scoring system of the International Society on Thrombosis and Haemostasis (ISTH) [28]. The change in fibrin/fibrinogen degradation product (FDP) was used as the fibrin-related marker for the ISTH criteria. FDP values of <10, ≥10 but <25, and ≥25 mg/L, were defined as no increase, moderate increase, and strong increase, respectively. The outcome measure was the 28-day and hospital all-cause mortality.

### Body temperature

T_b_ recorded within 24 h of a diagnosis of severe sepsis was used for the APACHE II score and the recorded temperature was used in this analysis. We recorded the value measured by the method most preferred by the American College of Critical Care Medicine and the Infectious Diseases Society of America [2]. Although the method used to measure core T_b_ was not standardized and the specific methods used for each individual measurement of core T_b_ was not recorded in this survey, all the institutions that participated in this study used standard methods for determining core T_b_. The sites used for T_b_ measurement at the institutions were as follows: urinary bladder, ten institutions; urinary bladder or rectal, three institutions; rectal, one institution; and intravascular, one institution. To ascertain the effect of T_b_ aberrance on disease severity and outcome, patients were grouped into one of six categories based on their core T_b_ as recorded for the APACHE II scoring. The categories were ≤35.5°C, 35.6 to 36.5°C, 36.6 to 37.5°C, 37.6 to 38.5°C, 38.6 to 39.5°C, and ≥39.6°C, as previously reported. A core T_b_ of 35.5°C was taken as the lowest T_b_ value because previous studies have reported this temperature as the threshold of hypothermia with high mortality [16,29-34]. Although previous studies considered 35.5°C as the threshold for hypothermia [16,29-32], we opted to use 36.5°C as the threshold because we found significant differences in the mortality of patients with T_b_ ≤36.5°C compared with those with T_b_ >36.5°C based on the results of a temperature categorical analysis, as shown in Tables 1 and 2. We also compared the mortality of patients divided into two groups using 36.5°C as a cutoff value. This analysis demonstrated significant differences between groups in both the 28-day and hospital mortality (*P* <0.001). Based on these findings, we evaluated the effect of hypothermia defined as T_b_ ≤36.5°C on not only mortality but also disease severity, which may affect mortality. For analysis, patients were divided into two groups, namely, hypothermia (T_b_ ≤36.5°C, n = 160) and no-hypothermia (T_b_ >36.5°C, n = 464).

**Table 1:** Baseline characteristics and outcome of the enrolled patients (n = 624)

**Characteristic**	**Value**
Age, years	72 (61 – 81)
Gender, male/female	391/233
APACHE II score	23 (17 – 29)
SOFA score	8 (6 – 11)
MODS, n (%)	144 (23.1)
Septic shock, n (%)	282 (45.2)
Admission category	
Medical	519 (83.2%)
Trauma	27 (4.3%)
Surgery	19 (3.0%)
Burns	19 (3.0%)
Other	40 (6.4%)
Site of Infection	
Pulmonary	261 (41.8%)
Intra-abdominal	133 (21.3%)
Urinary	78 (12.5%)
Skin/soft tissue	78 (12.5%)
Meningitis	15 (2.4%)
Catheter-related	11 (1.8%)
Bone/joint	10 (1.6%)
Infective endocarditis	3 (0.5%)
Other	25 (4.0%)
28-day mortality, n (%)	144 (23.1%)
Hospital mortality, n (%)	184 (29.5%)

Values are presented as median (IQR) or number (%). APACHE, acute physiology and chronic health evaluation; SOFA, sequential organ failure assessment; MODS, multiple organ dysfunction syndrome.

**Table 2:** Body temperature and severity of coagulation abnormality/organ failure scores

	**Body temperature, °C**					
	**≤35.5 (n = 99)**	**35.6 to 36.5 (n = 61)**	**36.6 to 37.5 (n = 112)**	**37.6 to 38.5 (n = 155)**	**38.6 to 39.5 (n = 133)**	**≥39.6 (n = 64)**
Age, years	76 (65 to 82)^de^	78 (62.5 to 83.5)^de^	75 (66 to 84)^de^	72 (61 to 82)^de^	67 (58 to 76)	65 (45.25 to 78.75)
Septic shock, n (%)	62 (62.6%)^bcde^	33 (54.1%)	46 (41.1%)	63 (40.6%)	54 (40.6%)	24 (37.5%)
SIRS criteria	4 (3 to 4)^abc^	3 (2 to 4)^bde^	3 (2 to 3)^cde^	3 (3 to 4)^de^	4 (3 to 4)	4 (3 to 4)
DIC score	5 (2 to 6)^c^	4 (2 to 6)	4 (2 to 5)	3 (2 to 5)	3 (2 to 5)	3 (2 to 5)
DIC ≥5, n (%)	28 (28.3%)^cde^	15 (24.6%)^c^	22 (19.6%)	20 (12.9%)	21 (15.8%)	7 (10.9%)
SOFA score	10 (7 to 12)^cde^	10 (7 to 13)^cde^	8 (5 to 11)	8 (5 to 11)	7 (5 to 10.75)	7 (6 to 10)
MODS, n (%)	35 (35.4%)^cde^	23 (37.8%)^cde^	27 (24.1%)	32 (20.6%)	23 (17.3%)	10 (15.6%)
APACHE II	28 (23 to 33)^bcde^	24 (20 to 29)	21 (16.25 to 27)	21 (16 to 27)	22 (17 to 26)	22 (17.25 to 30)
Outcome						
28-day mortality, n (%)	40 (40.4%)^bcde^	21 (34.4%)^cde^	23 (20.5%)	28 (18.1%)	21 (15.8%)	11 (17.2%)
Hospital mortality, n (%)	52 (52.5%)^bcde^	27 (44.3%)^bcde^	27 (24.1%)	39 (25.2%)	26 (19.5%)	13 (20.3%)

SIRS, systemic inflammatory response syndrome; DIC, disseminated intravascular coagulation; SOFA, sequential organ failure assessment; MODS, multiple organ dysfunction syndrome; APACHE, acute physiology and chronic health evaluation. ^a^*P* <0.0033 (after Bonferroni correction) versus 35.6 to 36.5°C; ^b^versus 36.6 to 37.5°C; ^c^versus 37.6 to 38.5°C; ^d^versus 38.6 to 39.5°C; ^e^versus ≥39.6°C.

### Assessments

Blood was collected at the time of admission to ICU and then daily thereafter as part of the routine clinical and laboratory tests using established standard laboratory techniques. Platelet counts and coagulation variables necessary to diagnose DIC were collected and APACHE II, SOFA, and DIC scores were assessed.

### Statistical analysis

Data are expressed as medians and interquartile ranges. All statistical analyses were performed using SPSS 19.0 for Windows (SPSS, Chicago, IL, USA). Comparisons between the 2 groups were performed using the Mann-Whitney’s U test, and categorical variables were summarized using proportions and compared between groups using either the Pearson’s chi-square or Fisher’s exact test, where appropriate. Kruskal-Wallis one-way analysis of variance and multiple chi-square tests were used for comparisons between multiple groups, and *P*-values were adjusted with the Bonferroni correction for multiple testing.

Odds ratios (OR) are reported relative to a reference range of T_b_, as previously reported [33]. We defined the reference range here as the T_b_ category of 36.6 to 37.5°C. Additionally, survival curves were derived by the Kaplan-Meier method and compared by the log-rank test for each range. We used a multivariate logistic model to assess the relationships between 28-day mortality and independent variables in patients with severe sepsis. Outcome (dead, 1; survived, 0) was used as the criterion variable, and age, gender (male or female), admission category of underlying medical condition (medical or other cause), SOFA score, APACHE II score, positive blood culture (yes or no), the presence of comorbidity (yes or no), and hypothermia (T_b_ ≤36.5°C or >36.5°C) were used as explanatory variables. Results are reported as OR, *P*-values, and 95% CI. Differences with a *P*-value <0.05 were considered to be statistically significant. Furthermore, *P* <0.0033 (after Bonferroni correction) was used for comparisons between groups in multiple testing (Table 2).

## Results

### Baseline characteristics and patient outcome

During the 1-year study period, a total of 14,417 patients were admitted to the 15 critical care centers, and 624 (4.3%) of these patients were diagnosed with severe sepsis and enrolled in this study. The characteristics at enrollment and outcomes of patients are shown in Table 1. The mean age was 69 years, and the mean initial APACHE II score and SOFA scores were 23.4 and 8.6, respectively. The major sites of infection were pulmonary, intra-abdominal, urinary, and skin/soft tissue. More than half of the patients had dysfunction of three or more organ systems. The 28-day mortality was 23.1% and the overall hospital mortality was 29.5%. Sepsis-related hospital mortality was 25.6% (160/624 patients).

### Relationships between body temperature and severity scores

Patients with T_b_ >38.5°C were significantly younger than patients with T_b_ ≤38.5°C. The prevalence of septic shock was significantly higher among patients with T_b_ ≤35.5°C when compared with the incidence of septic shock among patients in the other T_b_ categories (Table 2). MODS and SOFA on the day of enrollment were significantly higher in patients with T_b_ 35.6 to 36.5°C and ≤35.5°C when compared with patients who had T_b_ >37.5°C. The APACHEII scores in patients with T_b_ ≤35.5°C were significantly higher when compared with patients who had T_b_ of >36.5°C.

For mortality rates, patients who had T_b_ ≤36.5°C had significantly higher 28-day and hospital mortality rates when compared with patients who had T_b_ >36.5°C. The mortality rate among patients who had T_b_ ≤35.5°C was especially high at 40.4% and 52.5% for 28-day and hospital mortality rates, respectively. The lowest 28-day and hospital mortality were noted in patients with T_b_ between 38.6 and 39.5°C (15.8% and 19.5%, for 28-day and hospital mortality, respectively) (Table 2).

### Body temperature and mortality

**Figure 1:**
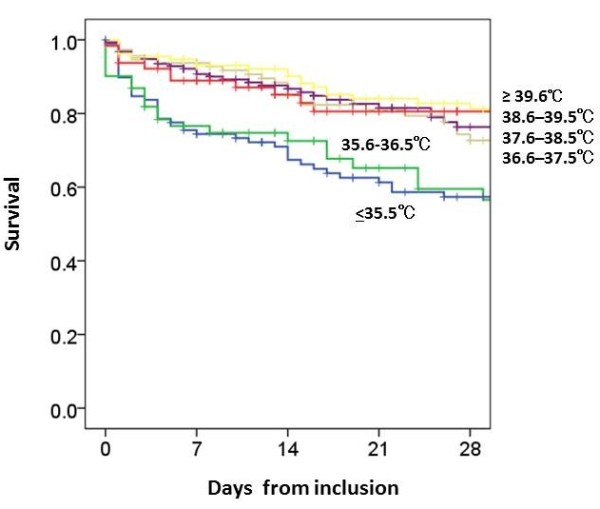
**Body temperature within 24 h of ICU admission and survival of patients with severe sepsis.** This figure depicts the Kaplan-Meier estimates for the probability of survival, which at 28 days was lower in patients with body temperature of ≤35.5°C and 35.6 to 36.5°C, as compared to patients with body temperatures of 36.6 to 37.5°C, 37.6 to 38.5°C, 38.6 to 39.5°C, and ≥39.6°C (*P* <0.001). Body temperature was recorded as the highest score on the acute physiology and chronic health evaluation (APACHE) II scoring system and as the farthest value from 36.5 to 37.0°C within 24 h from the time of enrollment, which was divided into categorical variables with 1°C increments. Thus, body temperature was analyzed in six range categories: ≤35.5°C, 35.6 to 36.5°C, 36.6 to 37.5°C, 37.6 to 38.5°C, 38.6 to 39.5°C, and ≥39.6°C.

Table 3 shows 28-day mortality and OR for each T_b_ (taken on day 1) relative to the reference range of 36.6 to 37.5°C. We found no relationships between mortality and T_b_ in patients in the following categories: 37.6 to 38.5°C, 38.6 to 39.5°C, and ≥39.6°C. The relationship between mortality and T_b_ was significant in patients in the T_b_ categories of 35.6 to 36.5°C (OR 2.032, *P* = 0.047) and ≤35.5°C (OR 3.096, *P* = 0.001). Kaplan-Meier estimates for the probability of survival at 28 days were lower in patients with T_b_ between 35.6°C and 36.5°C and ≤35.5°C compared to patients who had T_b_ ≥36.6°C (Figure 1).

**Table 3:** Day-1 body temperature and 28-day mortality

**Range of body temperature (°C)**	**28-day mortality**	**Unadjusted odds ratio**	**95% CI**	***P*-value**
≤35.5	40.4%	3.096	1.611, 5.947	0.001
35.6 to 36.5	34.4%	2.032	1.009, 4.088	0.047
36.6 to 37.5	20.5%	1.000	(reference)	
37.6 to 8.5	18.1%	0.853	0.461, 1.577	0.621
38.6 to 39.5	15.8%	0.726	0.377, 1.395	0.404
≥39.6	17.2%	0.803	0.363, 1.778	0.693

### Severity scores and outcome in hypothermic and non-hypothermic patients

After analyzing the data for the different T_b_ categories, we defined 36.5°C as the threshold temperature for hypothermia and compared variables and outcomes between patients with hypothermia (≤36.5°C, n = 160) and those without hypothermia (>36.5°C, n = 464).

The incidence of septic shock was significantly higher in patients with hypothermia compared to patients without hypothermia. DIC, SOFA, and APACHE II scores and the incidence of MODS were significantly increased among hypothermic patients (Table 4). In hypothermic patients, 28-day and hospital mortality were higher (more than double) than the mortality rates of patients without hypothermia (38.1% versus 17.9%, 49.4% versus 22.6%, for 28-day and for hospital mortality, respectively) (Table 4).

**Table 4:** **Characteristics, physiology on day 1 and outcome in hypothermic (body temperature ≤36.5**°C**) and non-hypothermic severe sepsis patients**

	**Hypothermia (n = 160)**	**Non-hypothermia (n = 464)**	***P*-value**
Age, years	76 (64.25 to 83)	71 (60 to 80)	0.001
Septic shock	59.4% (n = 95)	40.3% (n = 187)	<0.001
DIC score	4 (2 to 6)	3 (2 to 5)	0.009
SOFA score	10 (7 to 13)	8 (5 to 11)	<0.001
APACHE II score	26 (21 to 32)	21 (16.25 to 27)	<0.001
Outcome			
28-day mortality	38.1% (n = 61)	17.9% (n = 83)	<0.001
Hospital mortality	49.4% (n = 79)	22.6% (n = 105)	<0.001

Results are presented as median (IQR) or % (number). DIC, disseminated intravascular coagulation; SOFA, sequential organ failure assessment; APACHE, acute physiology and chronic health evaluation.

### Comparisons of severity scores and outcome between hypothermic and non-hypothermic patients with and without septic shock

The incidence of septic shock was significantly higher in patients with hypothermia. We separately evaluated the influence of hypothermia on variables and outcomes in patients with and without septic shock, because mortality and severity scores in patients with septic shock were significantly higher when compared with other patients [23]. Patients with septic shock had higher DIC, SOFA, and APACHE II scores if they were hypothermic at the time of diagnosis. In these hypothermic patients, both 28-day and hospital mortality were nearly twice those of patients with septic shock and no hypothermia (Table 5).

**Table 5:** **Characteristics, physiology on day 1, and outcome in hypothermic (body temperature ≤36.5**°C**) and non-hypothermic patients with septic shock**

	**Hypothermia (n = 95)**	**Non-hypothermia (n = 187)**	***P*-value**
Age, years	75 (62 to 83)	72 (61 to 79)	0.069
DIC score	4.0 (2.0 to 5.0)	3.0 (2.0 to 5.0)	0.047
SOFA score	11.0 (9.0 to 13.0)	10.0 (8.0 to 13.0)	0.039
APACHE II score	29.0 (23.0 to 35.0)	25.0 (19.0 to 31.0)	0.001
Outcome			
28-day mortality	49.5% (n = 47)	24.6% (n = 46)	< 0.001
Hospital mortality	62.1% (n = 59)	31.0% (n = 58)	< 0.001

Results are presented as median (IQR) or % (number). DIC, disseminated intravascular coagulation; SOFA, sequential organ failure assessment; APACHE, acute physiology and chronic health evaluation.

In patients without septic shock, hypothermic patients had a significantly higher incidence of MODS and they also had significantly higher SOFA and APACHE II scores when compared with patients who did not have hypothermia. Although the 28-day mortality was not significantly different between hypothermic and non-hypothermic patients, hospital mortality in hypothermic patients was nearly twice that of non-hypothermic patients (30.8% versus 17.0%) (Table 6). The 28-day and hospital mortality rates in hypothermic patients without septic shock and non-hypothermic patients with septic shock were both double those in patients who were non-hypothermic and did not have septic shock. In addition, the 28-day and hospital mortality rates in hypothermic patients with septic shock were almost four times higher than those in non-hypothermic patients without septic shock.

**Table 6:** **Characteristics, physiology on day 1, and outcome in hypothermic (body temperature ≤36.5**°C**) and non-hypothermic patients without septic shock**

	**Hypothermia (n = 65)**	**Non-hypothermia (n = 277)**	***P*-value**
Age, years	78 (70 to 82.5)	71 (58 to 81)	0.004
DIC score	3 (2 to 5)	3 (2 to 5)	0.133
SOFA score	7.5 (5 to 11)	6 (4 to 8)	0.004
APACHEII score	24 (18 to 27)	19 (15 to 24)	<0.001
Outcome			
28-day mortality	21.5% (n = 14)	13.4% (n = 37)	0.096
Hospital mortality	30.8% (n = 20)	17.0% (n = 47)	0.012

DIC, disseminated intravascular coagulation; SOFA, sequential organ failure assessment; APACHE, acute physiology and chronic health evaluation. Results are presented as median (IQR) or % (number).

Table 7 shows that hypothermia, defined as a core T_b_ of ≤36.5°C, was an independent predictor of 28-day mortality in patients with severe sepsis, especially in the presence of septic shock.

**Table 7:** Results of multivariate logistic regression analysis for the prediction of 28-day mortality

**Factors**	**Odds ratio**	***P*** **value**	**95% CI**
Severe sepsis (n = 602)			
Age	1.026	0.001	1.010–1.042
Gender (male)	1.476	0.091	0.940–2.317
Admission category (medical conditions)	1.098	0.778	0.572–2.109
SOFA score	1.111	0.002	1.041–1.186
APACHE II score	1.062	0.000	1.029–1.095
Positive blood culture	1.471	0.073	0.965–2.242
Presence of comorbidity	0.880	0.569	0.566–1.367
Hypothermia (body temperature <36.5°C)	1.952	0.003	1.253–3.040
Severe sepsis with septic shock (n = 273)			
Age	1.036	0.001	1.014–1.059
Gender (male)	1.676	0.095	0.914–3.074
Admission category (medical conditions)	1.110	0.829	0.431–2.860
SOFA score	1.078	0.119	0.981–1.186
APACHE II score	1.050	0.019	1.008–1.094
Positive blood culture	1.761	0.052	0.996–3.114
Presence of comorbidity	0.722	0.290	0.395–1.319
Hypothermia (body temperature <36.5°C)	2.778	0.001	1.555–4.965

CI, confidence interval; SOFA, Sequential Organ Failure Assessment; APACHE, Acute Physiology and Chronic Health Evaluation; Comorbidity, at least one comorbidity.

## Discussion

The results of this study clearly indicate that the mortality rate amongst patients with severe sepsis is significantly higher among those who have a T_b_ of ≤36.5°C compared to those who have a T_b_ of >36.5°C measured within 24 h of diagnosis. In this study, the mortality rate was more than two times higher among patients with severe sepsis who were hypothermic compared to patients with severe sepsis who had no hypothermia. Furthermore, the higher mortality rate was associated with a deterioration of organ function and DIC. The effect of hypothermia on mortality rate was consistently observed in patients with and without septic shock.

In our study, elevated T_b_ was not associated with an increase in disease severity or risk of mortality. Moreover, elevated T_b_ was not associated with a progressive increase in disease severity or mortality when compared with the reference T_b_ range of 36.6 to 37.5°C. Our results suggest that higher T_b_ is not harmful in patients with severe sepsis. Studies investigating the effect of fever control by means of antipyretic treatment or external cooling and the risk of mortality have reported contrasting results, and it is clear that the role of fever and its control in patients with severe sepsis still needs to be elucidated [34,35].

It has been suggested that hypothermia is associated with an increased risk of mortality in critically ill patients [12,33]. Moreover, the effect of hypothermia on increased mortality has been shown in patients with and without infection [12,17,18]. In the Methylprednisolone Severe Sepsis Study database, the Veterans Administration Systemic Sepsis Cooperative Study of Glucocorticoid Therapy, and the Ibuprofen Sepsis Study, the threshold T_b_ for hypothermia was set at 35.5°C and patients with severe sepsis were included. The incidence of hypothermia (<35.5°C), 28- or 30-day mortality in patients with hypothermia versus patients without hypothermia in these studies were 9%, 62% versus 26%; 10%, 57% versus 28%; and 9.6%, 70% versus 35%, respectively. The NORASEPT II study included only patients with septic shock and the incidence of hypothermia among these patients was 21%. The mortality in patients with hypothermia and in those without hypothermia was 59% and 34%, respectively. In the present study, the incidence of mortality among patients with T_b_ ≤35.5°C was 15.9% (99/624 patients), and the 28-day and hospital mortality rates were also significantly higher when compared with other patients (T_b_ >35.5°C; 40.4% versus 19.8%, 52.5% versus 25.1%, for 28-day and hospital mortality, respectively). Although the underlying mechanism of sepsis-related hypothermia is still unclear, our results are consistent with previous studies [16,29-32].

We defined 36.5°C as the threshold of hypothermia based on the results of our evaluation of the outcomes for the different T_b_ categories. We also generated the receiver operating characteristic curves using T_b_ on the day of enrollment for the 28-day and hospital mortality evaluation. The analysis revealed that the cutoff values for predicting the 28-day and hospital mortality were 36.9°C and 36.3°C, respectively, for maximizing both sensitivity and specificity (data not shown), and these suggest a T_b_ of 36.5°C as an acceptable cutoff value to define hypothermia in this study.

Although the impact of hypothermia, defined as a threshold temperature of 35.5°C, on mortality has been demonstrated in previous studies [16,29-32], the effect of hypothermia on disease severity has not been fully evaluated. Therefore, we evaluated the effects of T_b_ ≤36.5°C on both mortality and disease severity by comparing patients with and without hypothermia. The incidence of organ failure, DIC and outcomes were significantly different in patients with T_b_ of ≤36.5°C compared to those with T_b_ of >36.5°C, and there was no significant difference between patients who had T_b_ of ≤35.5°C compared to those with a T_b_ of >35.5°C. Therefore, 36.5°C was considered the threshold for hypothermia in patients with severe sepsis, irrespective of the presence of septic shock.

It is important to note that the inclusion of T_b_ abnormalities as a measure of the severity of illness varies between different scoring systems. APACHE II assigns points for patients with either high or low T_b_, SAPS II only assigns points for high T_b_, and SAPS III only assigns points for low T_b_[26,36,37]. Although it is widely accepted that fever has an adverse effect on patients with neurologic injury [38], little is known about the impact of temperature abnormalities on the outcome of other ICU patients, especially patients with sepsis [1,18]. The results of this study add valuable knowledge with regard to the influence of T_b_ abnormalities on the outcome of patients with severe sepsis. From our results, it is clear that hypothermia has a greater impact on organ dysfunction and outcomes. Thus far, the mechanism underlying the harmful effects of hypothermia is not yet known, but it is evident that hypothermia is more important than elevated temperature for the severity of illness scores in patients with severe sepsis.

### Limitations

There are some limitations to our study. T_b_ was recorded within 24 h of a diagnosis of severe sepsis as the highest core T_b_ value of the APACHE II score. However, we did not standardize the method by which core T_b_ was measured, and we did not attempt to differentiate between patients who had an elevated T_b_ as a result of hyperthermia syndrome or because of fever. Although some patients might have been categorized differently if we employed a systematic protocol for measuring T_b_, our recorded core T_b_ data were not merely arbitrary values obtained within the 24-h period after a diagnosis of severe sepsis, but these measurements were assessed objectively. In addition, the outcome and influence of treatment may vary significantly on that basis.

We did not specifically control for therapeutic modalities that may have influenced T_b_, such as antipyretic drugs or active external temperature control strategies. Although it is widely accepted that temperature control improves outcome in patients with neurologic injury, the effect of acetaminophen, ibuprofen, or external control on the outcome of other critically ill patients is not well understood.

## Conclusions

T_b_ of patients with severe sepsis, as measured at the time of diagnosis, significantly affected patient outcome. In our study, hypothermia (≤36.5°C) was associated with a significantly higher risk of mortality. The risk of mortality was almost double among hypothermic patients compared to patients without hypothermia. Hypothermia was also associated with a significant physiological decline in these patients, irrespective of whether they experienced septic shock or not. Elevated T_b_ was not associated with an increased disease severity and risk of mortality.

## Key messages

•In patients with severe sepsis, the impact of elevated body temperature and hypothermia on mortality and severity of physiologic decline is different.

•Hypothermia, defined as body temperature of ≤36.5°C, is significantly associated with an increased mortality risk of more than double that of non-hypothermic patients; moreover, it is associated with a physiological decline in severe sepsis, irrespective of the presence of septic shock.

•Elevated body temperature was not associated with increased disease severity or risk of mortality.

## Abbreviations

APACHE: Acute physiology and chronic health evaluation; DIC: Disseminated intravascular coagulation; FDP: Fibrin/fibrinogen degradation product; ISTH: International Society on Thrombosis and Haemostasis; JAAM: Japanese Association for Acute Medicine; JAAMSR: Japanese Association for Acute Medicine Sepsis Registry; MODS: Multiple organ dysfunction syndrome; OR: Odds ratio; SAPS: Simplified acute physiology score; SIRS: Systemic inflammatory response syndrome; SOFA: Sequential organ failure assessment; Tb: Body temperature.

## Competing interests

The authors declare that they have no competing interests.

## Authors’ contributions

SK participated in study design and data collection and interpretation, performed the statistical analysis, and drafted the manuscript. SG, DS, HO, NT, SF, TM, TA, HI, JK, YM, SS, KS, YS, KT, RT, YY, NY, and NA participated in study design and data collection and interpretation, performed the statistical analysis, and helped to draft the manuscript. All authors read and approved the final version of the manuscript.
